# Development of A High Throughput Method Incorporating Traditional Analytical Devices

**DOI:** 10.6028/jres.109.034

**Published:** 2004-10-01

**Authors:** C. C. White, E. Embree, W. E Byrd, A. R. Patel

**Affiliations:** National Institute of Standards and Technology, Gaithersburg, MD 20899-8621

**Keywords:** analytical chemistry, combinatorial, high efficiency, high throughput, methodology

## Abstract

A high-throughput (high throughput is the ability to process large numbers of samples) and companion informatics system has been developed and implemented. High throughput is defined as the ability to autonomously evaluate large numbers of samples, while an informatics system provides the software control of the physical devices, in addition to the organization and storage of the generated electronic data. This high throughput system includes both an ultra-violet and visible light spectrometer (UV-Vis) and a Fourier transform infrared spectrometer (FTIR) integrated with a multi sample positioning table. This method is designed to quantify changes in polymeric materials occurring from controlled temperature, humidity and high flux UV exposures. The integration of the software control of these analytical instruments within a single computer system is presented. Challenges in enhancing the system to include additional analytical devices are discussed.

## 1. Introduction

This paper describes the successful integration of two quantitative analytical instruments from different manufacturers into a high throughput screening system for quantitative characterization and analysis of a library of specimens subjected to a range of exposure regimes. The successful integration of these techniques into a high efficiency system illustrates the potential for integrating additional analytical devices in the future.

The application of high efficiency methods to scientific inquiry has enabled new approaches to long-standing scientific problems [1–15]. Significant progress on scientific problems previously considered intractable has been made through the combined use of intelligent, scientifically based experimental design, high efficiency methods and a underlying informatics system.

High efficiency methods consist of a combinatorial method and a high throughput method. Combinatorial methods generate large systematic libraries of samples or compounds that contain all possible combinations of the components of interest. High throughput methods are the companion techniques that use a sequential process to evaluate or measure the properties of samples in the combinatorial library.

The early pioneers of these approaches relied on a systematic approach to solving significantly large problems. For example, Gregor Mendal [16], considered the father of classical genetics, spent seven years carefully observing thousands of genetic crossings of the 22 strains of *Pisum sativum* [17]. Thomas Edison [18] also relied on testing thousands of materials before arriving at the carbonized cotton thread [19].

Significant improvements to these early systematic methods were achieved by increasing the use of automation and the incorporation of modern experimental design. Modern experimental design as part of high efficiency methods has been well documented elsewhere [20–24]. These design techniques greatly enhance the effectiveness of high efficiency methods by directing and focusing the combinatorial libraries. The use of design tools has enabled three to four decade increases in efficiency. This increase in efficiency enables complicated or massive problems to be addressed effectively. Sequencing the entire human genome is an example of one such a problem. The massive effort was enabled through four distinct high efficiency tools: efficient experimental design, targeted restriction enzymes building combinatorial libraries, high throughput automated gene sequencers and computer based informatics. The restriction enzymes and the automated gene sequencers are the physical tools that enable digital creation of large volumes of electronic data. The computer based informatics system is the ability to collect, store, manipulate and process the large volume of electronic data. The efficient experimental design then focuses the entire effort. It is the convergence of these four components: an efficient experimental design, targeted restriction enzymes, high throughput automated gene sequencers, and a powerful computer based informatics systems that enabled the entire human genome to be sequenced and reconstructed in less than 10 years. This milestone has sparked a revolution in most biology related scientific disciplines.

Despite this genomic accomplishment and other successes however, high throughput methods are generally limited to the automation of a single quantitative analytical device. Quantitative analytical devices, typically running proprietary software control are considered in stand-alone or all-in-one configurations.

As discussed previously, high throughput systems involving larger volume or solid samples are designed around a single analytical technique. For some problems, analysis by at least two analytical techniques is desirable or required. This seemingly simple step would greatly expand the range of possible scientific problems that could be addressed with a high efficiency solution. While the potential benefits are easily envisioned, the research community has been slow in realizing this potential [18]. Two generic barriers to the integration of multiple analytical devices also encumber the development of these high efficiency systems: the lack of ability to control instruments outside of the proprietary programs and the lack of development of integrated informatics schemes. Both of these major barriers directly stem from the traditional development of scientists and instruments. As the scientific community undertakes larger and more complex problems, the need for robust, high performance analytical devices that can be integrated together will grow. Meeting this demand requires scientists with a specialty in the integration of multiple techniques and specialization in more than a single technique. It also requires instruments designed for integration. These instruments will allow instrumental control by either the development of standard interface commands and interactive standards or by allowing third parties to develop software controls. Several different groups are working towards standard interfaces for laboratory automation [25,26]. There are also several third party programs that advertise such control over common analytical instruments. Thermo Galactic’s^2^ grams with the My Instrument module and Labview are two examples. At the 2004 Pittcon meeting several approaches to multiple technique control were presented, with all of the examples involving an adaptation of proprietary software to include two techniques from the same manufacturer [27]. Off-the-shelf integrated informatics system will be more readily available in the coming years. Developments in commercial tracking systems used in business and biology will drive the development and evolution of these systems. With simple modifications, off the shelf versions can be available for these high efficiency systems. One exciting development in this area is the development of “zero-footprint” control software that uses web-based application. While still very experimental this approach would overcome the barriers presented by the use of proprietary software. To date this concept has not been widely adopted [27].

## 2. Service Life Prediction

The prediction of the in-service life of components designed for outdoor use is a problem that cannot be reasonably solved without the application of high efficiency techniques. These techniques are required to generate the volume of data necessary for evaluations in a reasonable amount of time. These data are then used to develop and verify predictive mathematical models of a material’s behavior.

### 2.1 Scope of the Problem

For every product designed for outdoor exposure, a critical question to consider is what is the in-service performance of that product. To answer this basic question a significant amount of work is required. For example, consider the case of a prototype coating for metal. A typical coating system may be composed of up to seven different components (e.g., base material, clear coat, UV additive packages, viscosity modifiers, base pigments, specialty pigments, tackifiers, etc.). The range of possibly significant factors affecting this coating during environmental exposure is potentially enormous. Despite greatly reducing the number of these factors, it will require a very large number of sample evaluations to understand the performance mechanisms of the candidate materials. Even with a fractional factorial experimental design, evaluating this number of combinations would be too expensive and time consuming without the use of high efficiency methods.

## 3. Application of High Efficiency Methods

Four convergent elements are required to implement a high efficiency method to the service life prediction problem: a dose-damage approach, high efficiency devices to produce the dosage, high throughput methods to quantify the damage, and a companion informatics system.

### 3.1 Dose-Damage Approach

Other scientific fields have successfully used mathematical models to relate a material’s mechanism of damage to the exposed environmental conditions. One of the best documented models relates the *damage* to the DNA of skin cells resulting from a *dose* of ultraviolet (UV) exposure. The accepted method of quantifying the dose of UV radiation is a double integral of the intensity of the light source multiplied by an absorption factor and a spectra quantum yield over both time and wavelength. This dosage relationship is not dependent on the rate of exposure, but rather on the total quanta of light exposed. This approach is well accepted in medicine and other biological fields. The use of this approach allows rapid accumulation of dose through exposure to high intensity light sources, which shortens the time required to evaluate materials. For this example, the dose consists of one factor: the accumulation of UV light. For other materials, this approach requires inclusion of other factors such as moisture and temperature into the basic dose accumulation calculation.

Quantifying the damage to the material is most effective if based on fundamental mechanistic knowledge. Fortunately, for most material systems this information is available and readily monitored with existing technology. An example of this type of mechanism is the understanding that a specific polymer coating degrades by chain scission. Detecting the change in the chemical composition through quantitative Fourier Transform Infra Red (FTIR) spectroscopy can monitor this chain scission. Examining these spectra allows for a quantitative molecular measure of the damage occurring in the material.

### 3.2 Producing a High Efficiency Controlled Dose

One of the major impediments to producing an accurate prediction of in-service performance is the time required to evaluate the product. Relying on “natural” exposure to generate the dose typically requires 26 months. With the application of a cumulative dosage model like that accepted for skin exposure, the dosage is related to the quanta of light and not the rate. While this assumption has to be experimentally verified for service life prediction of materials, it suggests a high throughput sample device for producing the dosage is possible. The following features would be attractive in such a device: a high flux, uniform stable source of UV, a shortened time required to produce the dose, the ability to uniformly expose a large number of samples at one time, and the ability to independently control the principle dosage factors. Such a device has been built at NIST (i.e., the NIST SPHERE: Simulated Photodegradation via High Energy Radiant Exposure) [29–31]. A complete description of this device is presented elsewhere, but a summary is presented here.

**Fig. 1:**
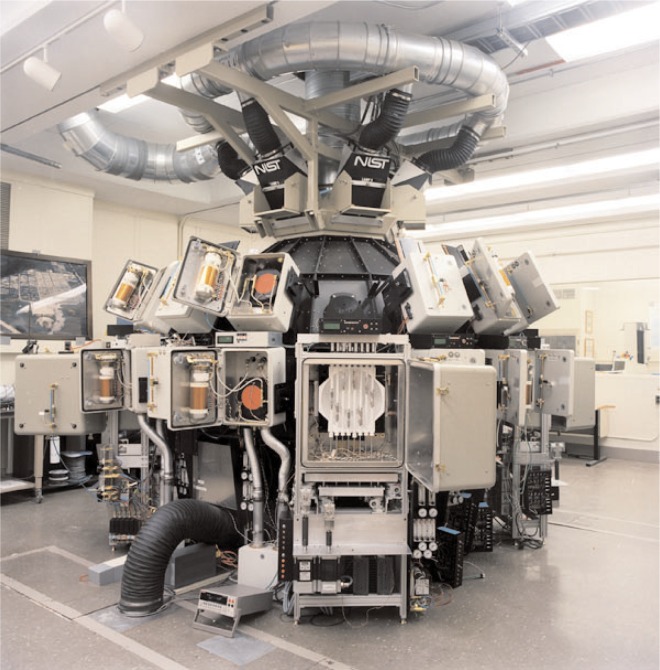
Photo of the NIST SPHERE (Simulated Photodegradation by High Energy Radiant Exposure). This device can expose over 500 samples in one of 32 individually controlled environments. Each environment is controlled with respect to temperature, humidity, Ultra-Violet Radiation. Each sample can receive up to 24 times the radiant flux of noonday sun.

**Fig. 2:**
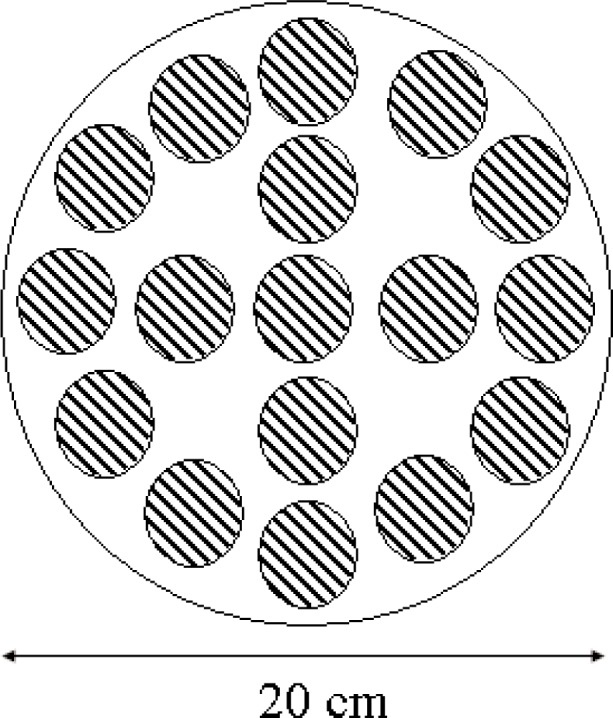
Configuration of the seventeen-sample, 20 cm diameter magazine. Each sample, shown as striped region is 19 mm in diameter.

This device, pictured in Fig. 1, is based around a 2 m integrating sphere that acts as a uniform high intensity, collimated UV light source where irradiant levels equivalent to approximately 24 “suns” [31] can be achieved. The temperature and relative humidity can be independently and precisely controlled over a wide range of values and also over long exposure periods for each of the 32 sample ports on the surface of the sphere. In the current experimental configuration, each port has 17 different sample positions (i.e. 544 total samples). These 17 samples are assembled in an aluminum body termed a magazine, which is illustrated in Fig. 2. The exposure conditions in each of the exposure chambers are constantly controlled and monitored using automated sensors for each of the principal environmental stresses: temperature, UV and humidity. This sensor data is stored in separate data files.

The NIST SPHERE is designed to generate a large combinatorial library of samples with different exposure histories. In the current configuration each of the 544 samples need to be daily characterized. This volume of data (1100–1500 measurements/day) makes the traditional application of standard techniques for analyzing the damage impractical. Instead, a custom, high efficiency method based upon well-established *analytical techniques* used in conjunction with an *automated sample positioning system* and a *centralized control system* is required to analyze the volume of dosage samples generated by the NIST SPHERE.

## 4. Screening Device to Rapidly Quantify the Damage

### 4.1 Analytical Techniques

Because environmental exposure will alter the polymeric material, two traditional methods are appropriate to characterize changes in the chemistry or damage: Fourier transform infra red (FTIR) spectroscopy and ultra violet/visible (UV/Vis) spectroscopy. These well established methods were selected based on the requirement that the methods be sensitive, quantifiable, non-destructive, rapid and suitable to automation. Both of these measurements can be performed in transmission through a clear sample or often in reflection of a pigmented system.

An FTIR (Nicolet Magna 560) spectrometer [27] equipped with an attenuated total reflectance (ATR) probe was used to obtain the chemical composition of the polymeric material. The ATR probe permits automated sample positioning external to the instrument and has the ability to measure spectra in reflection.

The second instrument, a Perkin-Elmer Lamda 900 UV-Vis spectrometer, was used to obtain the UV-Visible spectrum of the coating. This instrument was modified by the addition of an integrating sphere attachment produced by Labsphere. This attachment also allows for external sample positioning and has the ability to measure the reflective properties of polymeric materials. It is also capable of measuring the reflected spectra with and without the specular reflection component by means of a moveable light trap. The difference between the spectra with and without the specular reflection component is correlated to the surface gloss. This correlation is an added benefit because loss of coating gloss is one of the traditional methods of determining coating damage. Standard measurements of gloss are taken at 10°, 40°, and 70° normal to the surface, however the angle of incidence in the integrating sphere attachment to the UV/Vis spectrometer is 8 degrees. Furthermore, this technique produces an indirect measurement of gloss because the difference between the two spectra is compared. This high angle, indirect gloss measure will be most sensitive to the initial loss of gloss in very glossy samples.

The magazine assembly, illustrated in Fig. 2, has a diameter of 20 cm, is 2 cm thick, and has 17 sample openings that are each 19 mm in diameter. The magazine has a top plate and a bottom plate. The openings in the top plate are 1 mm in diameter smaller than that of the sample holder thus creating a small lip to prevent the samples from falling out through the top plate. The bottom plate is a solid aluminum disk. To load the samples, the bottom plate is removed and the samples are placed into the openings. After the samples have been loaded, a spring is added and the bottom plate is replaced. The samples are held in place in the magazine by the lip of the top plate and the spring on the bottom.

### 4.2 Automated Sample Positioning System

**Fig. 3:**
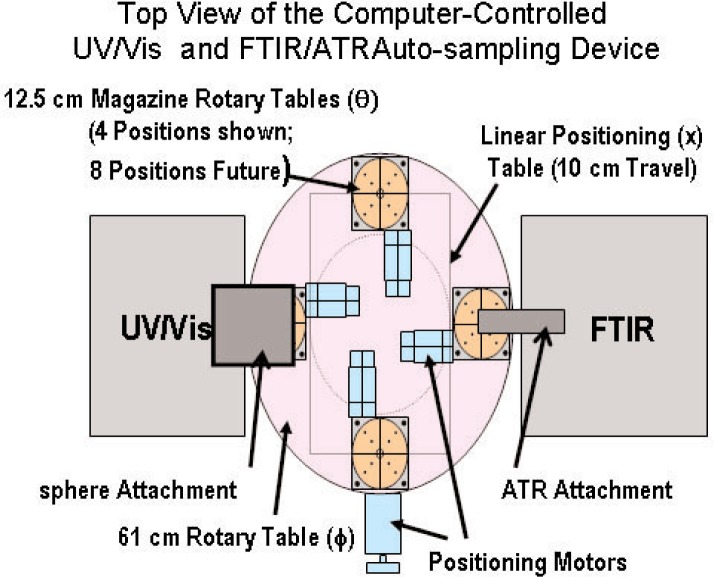
Top view of the screening device showing positions of FTIR spectrometer with ATR attachment and UV/Vis spectrometer with the Labsphere attachment are shown along with automated sample-positioning table with four magazine positions, a linear positioning system, and a 61 cm diameter rotatory table.

**Fig. 4:**
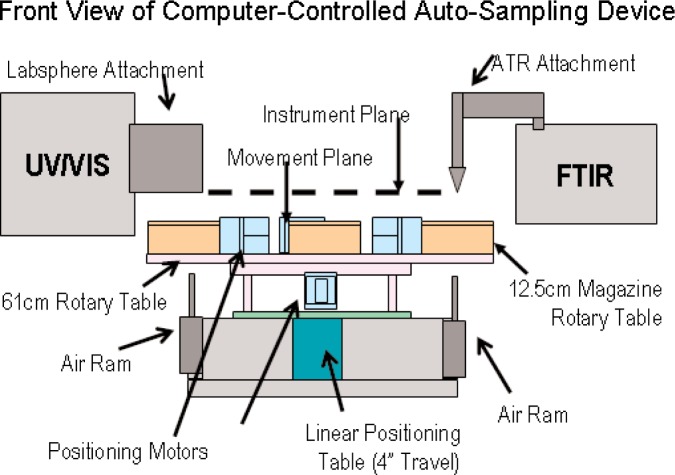
Front view of screening device in which the instrument plane and movement plane is identified. FTIR spectrometer with ATR crystal is positioned over center of magazine holder. This is also true for the sample port on Labsphere attachment to the UV/Vis spectrometer. This center position also contains the air rams also pictured.

**Fig. 5:**
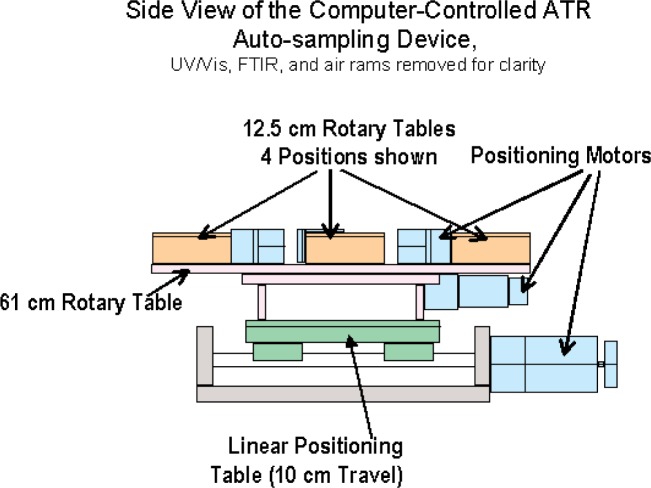
Side view of screening device. FTIR/ATR, UV/Vis/Labsphere, air rams and undercarriage side rails have been removed to highlight linear positioning system.

The purpose of an automated sample positioning system is to increase the efficiency of an instrument by allowing unattended measurements. For simplicity, the FTIR and UV/Vis spectrometers were positioned to face each other with the ATR and Labsphere attachments extending into the space between them (see Fig. 3). Also, the instruments are positioned so that the probes are in the same vertical plane referred to as the instrument plane (see Fig. 4). These two probes define the points of contact for the two instruments and are initially positioned over the center of a magazine assembly. The automated sample positioning system (see Figs. 3, 4, and 5) moves the magazine assembly in the movement plane located 3 cm below the instrument plane. By keeping the samples in the movement plane, they can be moved into position without damaging either the samples or the instrument sample probes. After positioning a sample directly below a spectrometer probe, the positioning system lifts the magazine assembly to bring the sample into contact with the probe.

There are three required movements to position any part of the magazine system directly under the instrument probe and bring a sample into a measurement position: the rotation of the magazine system (*θ*), the linear motion of the magazine system (*x*), and the vertical displacement (*z*) (see Fig. 3). First the instrument probes are positioned over the center of the magazine system. In vertical movement, the center position is brought into contact with the instrument. Once a measurement is executed, the magazine is lowered back to the movement plane. If the samples in the movement plane are then translated a distance in the *x* direction less than or equal to the radius of the magazine assembly, any part of a magazine rotary table radius can then be brought into contact with the instrument probe through a vertical displacement of the magazine. The entire magazine can thus be sampled through of a translation in the *x* direction followed by a rotation of the magazine assembly (*θ*) and finally, a vertical displacement (*z*) into the instrument plane followed by a vertical movement back into the movement plane. The 60 cm circular table can hold eight magazine assemblies. Once all the positions of the magazine have been measured, a simple rotation of the rotary table (*ϕ*) will move another sample magazine into place to be measured, and the entire magazine measurement process can be repeated.

To achieve a high degree of positional accuracy, Parker Compumotor stepper motors were used. For the linear *x* positioning, an 800CT table with a series 23 motor was used. For the rotation of the entire table (*ϕ*), a 60 cm diameter, 0.5 cm thick black anodized aluminum plate was mounted on a 30 cm rotary table, which was also powered by a series 23 motor. Each of the magazine rotation stages (*θ*) was mounted on a 200 series RT 125 mm diameter rotary table also driven by a series 23 motor. All of the motors were controlled and indexed with separate Zeta 6401 drive controller/indexers. The Zeta 6401 drive/indexers can be chained together allowing one RS232 serial connection address to control each stepper motor independently. The positional uncertainty of this system is a combination of the stated linear positional uncertainty ± 3 µm (*x*) plus the stated repeated positional uncertainty of the rotary stages, ± 5 µm (*θ*), ± 17 µm (*ϕ*) which totals to ± 25 µm. Because this uncertainty is much smaller than the size of the ATR probe tip, the system should be able to measure the same spot upon repeated positioning.

Vertical movement of a magazine rotary table is made through the action of a fixed position air ram. The pneumatic ram aligned with a hole in the rotation table (*ϕ*), lifts the sample magazine assembly into contact with the analytical instrument. For the UV/Vis instrument, the pressure of the air ram, which is material dependent, is sufficient to ensure that the sample is in contact with the integrating sphere. For the FTIR/ATR crystal, the pressure of this air ram is a more significant issue. Too much pressure and either the sample or ATR crystal will crack, too small a pressure and there is not good contact between the ATR crystal and the sample of interest. Currently, this pressure is set manually by gradually increasing the pressure until a good FTIR/ATR spectra is obtained. The ATR crystal requires enough pressure for good contact with the sample, and the pressure of the air ram can cause the ATR to indent into the sample. As the sample ages, the mechanical properties may change. The air rams are activated/deactivated by using one controllable output from the Zeta 6104 driver/indexer to open and close an air valve, thus pressurizing or depressurizing the air ram.

This auto sample positioning system currently allows four magazines to be loaded and analyzed during unattended operation. All of these devices are connected to a personal computer. Automated operation has been achieved through the development of custom control software as detailed in the next section.

### 4.3 Central Control System

The controlling software was developed with four basic requirements:

- Establish all timing and event sequence;
- Move the samples into contact with the instruments;
- Initiate measurement by each of the instruments;
- Retrieve and store the data.

Two proprietary platform languages on the Nicolet FTIR and Perkin Elmer UV/Vis spectrometers complicate these requirements. Neither language includes a provision for external control.

A master control program, written in Visual Basic, was developed to establish the timing and event sequencing, move the samples and initiate measurements. The proprietary platform languages of the spectrometers format and store the spectra scans in a designated folder and with a designated name on the local hard drive. The advantage of this strategy is that the master program allows direct control of the automated sample positioning system and can use external active X calls to initiate the proprietary platform languages. The stepper motors were controlled through the RS232 port including the accessory port for the air ram. The Nicolet FTIR was controlled through the proprietary interface card and the Perkin Elmer UV/Vis through a third RS232 serial port.

Once the samples are positioned in contact with the analytical instruments through a series of commands to the various stepper motors and air ram, an external call would be used to start the measurement on either the UV/Vis or the FTIR. These proprietary platform languages are not designed for autonomous operation and include various pop-ups windows that require input before proceeding. They also do not offer any direct method of status reporting. To overcome these restrictions, the visual basic master control program was programmed to look for these pop-up windows, provide the necessary input and then use these windows as indicators of the status of the measurement procedure. By monitoring and providing input to the proprietary platform languages, the master control program can direct the format, name and location of the generated data file. To indicate when the proprietary program has completed the data acquisition and is ready for the next sample, the visual basic program monitors the CPU usage. When the programs have completed the data collection and stored the results in the indicated file, the CPU usage drops considerably. Only when these two indicators—the final pop-up window and a drop in CPU usage—have been received does the master control visual basic program initiate the commands to move to the next sample.

The time required to obtain the FTIR and UV/Vis spectra is limited by the analytical instrument data acquisition time. There is a trade-off especially in the FTIR, between the number of scans, which is proportional to the signal to noise ratio, and the total data acquisition time.

The volume of data generated by the screening device to rapidly quantify the damage will quickly overwhelm any traditional attempts to deal with the data files. If we consider just the data generated by this device, each of the 544 samples will be characterized with 3 files per day, thus generating >1600 spectra files every day. Therefore a sophisticated informatics system is required to store, retrieve and perform secondary analysis of this volume of primary data.

## 5. Informatics System

An informatics system was developed to track all of the information relevant to each of the samples studied. This information comes from a variety of sources including text files containing notes about sample creation, spectra files associated with single time points, log files that track the progress of the sample, files that track the cumulative dosage and data additional files from other proprietary software platforms. All of these data files must be an integral part of the informatics system for this application. Additionally, the system should have the ability to:

- organize, format, and store the raw data;
- store a large number of samples rapidly with a minimum of human effort;
- treat the files the same, regardless of the type of datafile;
- retrieve and sort the data over any platform;
- perform automated secondary analysis on the raw data.

### 5.1 Database Design

Considering the range of possible data types, the data storage and retrieval system needed to be as generic as possible. A meta-data database design was selected. In this design, the various file types are kept in a central location and identified only by a simple identifier code. The overall database is then composed of a much simpler, limited field, uniform format database (i.e., the meta-data database) containing enough data to identify and characterize the various files stored in the central location along with the specific pointer to the files. This very straightforward design allows for rapid, centralized offsite storage and retrieval of a wide variety of data types by removing most of the bulk information into a generic storage area and keeping the much smaller sample identifier data within the database. The basic function is storage and retrieval of the data; however, more complicated data functions such as secondary data analysis can also be accomplished.

Secondary analysis is performed by searching the meta data for the relevant files, retrieving the relevant data and placing them into a set location such as a directory. The files for a particular sample will include all of the raw data files from a variety of instruments, the exposure sensor data, any secondary analysis files, log files and also data from additional instrumental scans. When all of this data is assembled into a directory, a customized secondary analysis can then be performed within this directory. The resulting data from the secondary analysis is then stored in a new data file and archived with the other raw data, and a corresponding meta-data record is created in the meta-data database.

### 5.2 Database Implementation

To implement this meta-data bulk storage solution, a Dell server with 100 Gb of storage, 1 Gb of RAM, dual 900 MHz processors running Microsoft Windows 2000 and SQL was used. This remotely located networked hardware was the principal storage for both the data files and the meta-data database. Microsoft Access was chosen for the meta-data database program for the speed and ease of implementation. This program runs on the centralized server. It can effectively handle the limited field meta-data database and—with the great number of preprogrammed database functions—allows for rapid implementation.

Choosing the number and complexity of the fields comprising the meta-data was a critical decision in database implementation. The fields chosen to make up the meta-data were magazine serial number, experiment name, sample name, substrate, material, created by, and date. These fields constitute the main table of information and allows for each of the magazines to be uniquely identified. Additionally, each magazine record has a sub-table with the following five fields: file name, wafer barcode, sample position, instrument and time point. The file name is used to indicate the name of the file in the bulk storage and was chosen based on a root name that identifies the magazine to which the sample belongs plus extensions to identify the specific time point. The barcode entry was selected to accommodate the future addition of using barcodes to track the magazines, look up the meta data and populate the input forms. The sample position field was chosen to pick up the position of the sample within the magazine. The instrument field helps identify the source of the data and hence, the type of file referred to by the filename. The time point field was chosen to help identify the particular sample. The combination of a main table for characterizing the magazine with a sub-table with the individual files pointers allows for rapid sorting of a large number of data entries.

### 5.3 Database Operation

The ultimate success of the database structure depends upon the ease, efficiency and accuracy of populating the informatics system with data, organizing the data, and outputting the requested data files. Inputting data into the meta-data database structure requires three tasks: creating the meta data records, renaming the data files and moving the data files from the local data computer to the centralized server. The first task—creating the meta data records—is accomplished by running a customized Microsoft Access form from the central database server over the network to the designated local data input computer. This form asks the user to input the required meta-data for each of the magazines. Following this step a customized visual basic program creates the appropriate table entries in the Microsoft Access meta-database including the names of all the files. The visual basic program then renames all of the spectra files on the local data acquisition computer and transfers the renamed files to the central database server through the use of active X calls. The transfer of all of the files is confirmed on the central database server. The user is then asked to remove the files from the local computer. This last step is critical, because the data acquisition computer is programmed to use the same file names and locations for a given magazine position. Failure to remove the files from the local computer would result in possible duplication or corruption of data. The ability to input the data is restricted to specific computer input points and specific users through logins requiring specified IP numbers, usernames and passwords to the central database computer.

Input to the database must be highly restricted, but open access to the data is required in addition to maintaining security and integrity. To enable these features, a secure web based interface to the Microsoft Access database was developed. Using this interface, anyone with access to the server can sort and extract the data of interest without the ability to alter the database. Existing infrastructure for web-based interfaces for searchable Microsoft Access databases significantly decreased the development time for this web-based deployment. However, the secure, customized interface still required significant programming in XML. The major difficulty was not in displaying the Microsoft Access meta-data but in transferring the files from the central database server to the user’s computer over the web-based interface. Adding the file transfer utility appletfile version 2.5, obtained from the Infomentum Corporation, solved this problem. This applet enables secure web based file transfers. Joining the Infomentum Appletfile to Microsoft Access required significant XML coding. The final result is an informatics system that has the ability to securely input, sort, save and retrieve a large volume of raw data.

**Fig. 6:**
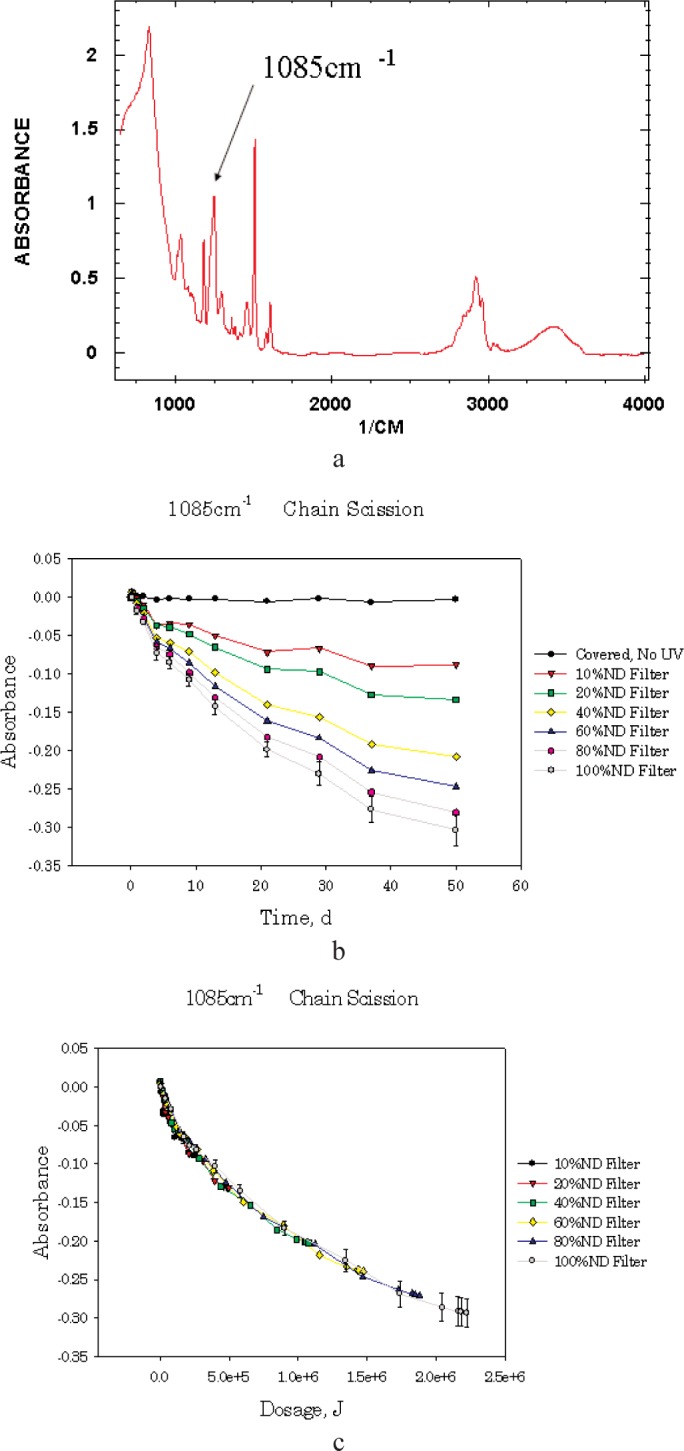
Example of the data stored and processed in the informatics system. Figure 6a shows a typical FTIR plot of an epoxy sample, the 1085 cm^–1^ peak is labeled. The secondary analysis program will read in each of these spectra, and determine the height of this peak. This data for is plotted as a function of time for seventy spectra in Fig. 6b. This experiment examines the impact of different neutral density filters from 100 % to 10 % transmission of the UV light. Figure 6c is the same information plotted as a function of dosage. Because all of the data collapse onto the same curve, the dose-damage assumption of the SPHERE is supported for this sample and experimental condition (25 °C ± 0.1 °C, 5 % ± 1 % relative humidity).

The secondary analysis of the raw data stored in the meta data database is accomplished by downloading the required data files from the file server onto a local computer through the web-based interface into a specific directory. An existing analysis program, developed specifically to handle large number of UV/Vis and FTIR spectra, is then used to automatically input the data files into the program, extract and process the raw spectra files and return a secondary analysis of the data. An example of this process is given in Fig. 6 for an epoxy system. This program processes hundreds of raw spectral files simultaneously, returning the damage versus time relationships for samples, magazines and collections of magazines. This screening distills the accumulated data from 1,600 files per day into three to four highly concentrated data files. These output data files are then loaded into the meta-data database. If future analysis indicates more study in a particular area is necessary, both the raw and processed data have been preserved and can be easily accessed and examined.

## 6. Future Improvements

Several areas where improvements to this system are planned include automation of the secondary analysis programs, direct control of the analytical instruments, bar code tracking of the magazines and direct measurement of the dose.

In addition, two major enhancements to the secondary analysis system are under development: the ability to use the actual dose and increasing system automation. In the current secondary analysis program, the user is required to input the measured set point dosages to which the samples were exposed. In the near future, direct measurement of the actual exposure conditions for the magazines will be stored in the meta data database and used in the secondary analysis of the samples. The second improvement to the informatics system outlined above will be automation of the secondary analysis program. In the present system, the secondary analysis program requires a user to sort and download the data and then manually run the secondary analysis program. This program will be shortly rewritten to be automatically deployed for each new experiment. These two innovations will significantly decrease the required, routine human involvement in the data analysis process.

A second major area of improvement is the direct control of the FTIR and UV/Vis. Currently, the master control program can call the proprietary platform language to begin a scan. The master control program will then wait and watch for the proprietary platform language to complete the scan and return the data. This is an indirect method of control. In a more robust system, all of the commands would be resident in the master control program.

The final area for improvement will be the addition of bar code tracking of the sample magazines. The existing system requires a user to recreate meta data for the magazine at every step. Plans are in place to use handheld wireless bar code scanners to scan bar codes on the exposure chambers, magazines and analysis table. The collected data will be used to automatically update log files, populate the meta data fields and greatly reduce the human involvement in the high throughput analysis system.

## 7. Summary

The successful development of a high throughput method incorporating traditional analytical techniques and companion informatics system are presented. FTIR and UVVis instruments are combined with a common automated sample-positioning table under a single computer control. This solution allows for rapid quantification of a large number of samples using multiple analytical measurement techniques. The example illustrated is that for service life prediction of materials exposed to outdoor environments.

During the development of this high throughput method incorporating traditional analytical techniques, many of the current generic difficulties in combining instruments from different manufactures are encountered and explored. Some of these difficulties resulted from the historical development of analytical instrumentation. This is typified by the belief that the instrument will have a dedicated computer running proprietary software solely interfaced to this one instrument. Possible avenues for removing or mitigating these difficulties are discussed. These avenues should help to catalyze the introduction of high efficiency methods to traditional analytical techniques. This infusion of analytical capability from these methods will enable significant progress in a wide range of large difficult problems such as the service life prediction for polymeric materials designed for outdoor exposure example presented here.

## Footnotes

2 Certain commercial equipment, instruments, or materials are identified in this paper to foster understanding. Such identification does not imply recommendation or endorsement by the National Institute of Standards and Technology, nor does it imply that the materials or equipment identified are necessarily the best available for the purpose.
